# Quercetin improves and protects Calu-3 airway epithelial barrier function

**DOI:** 10.3389/fcell.2023.1271201

**Published:** 2023-11-23

**Authors:** K. M. DiGuilio, E. Rybakovsky, M. C. Valenzano, H. H. Nguyen, E. A. Del Rio, E. Newberry, R. Spadea, J. M. Mullin

**Affiliations:** ^1^ The Lankenau Institute for Medical Research, Wynnewood, PA, United States; ^2^ The Departments of Biology and Chemistry, Drexel University, Philadelphia, PA, United States; ^3^ The Division of Gastroenterology, The Lankenau Medical Center, Wynnewood, PA, United States

**Keywords:** quercetin, flavonoid, tight junction, claudin, barrier function, tumor necrosis factor, cell division, Calu-3

## Abstract

**Introduction:** In light of the impact of airway barrier leaks in COVID-19 and the significance of vitamin D in COVID-19 outcomes, including airway barrier protection, we investigated whether the very common dietary flavonoid quercetin could also be efficacious in supporting airway barrier function.

**Methods:** To address this question, we utilized the widely used airway epithelial cell culture model, Calu-3.

**Results:** We observed that treating Calu-3 cell layers with quercetin increased transepithelial electrical resistance while simultaneously reducing transepithelial leaks of 14C-D-mannitol (Jm) and 14C-inulin. The effects of quercetin were concentration-dependent and exhibited a biphasic time course. These effects of quercetin occurred with changes in tight junctional protein composition as well as a partial inhibition of cell replication that resulted in decreased linear junctional density. Both of these effects potentially contribute to improved barrier function. Quercetin was equally effective in reducing the barrier compromise caused by the pro-inflammatory cytokine TNF-α, an action that seemed to derive, in part, from reducing the elevation of ERK 1/2 caused by TNF-α.

**Discussion:** Quercetin improved Calu-3 barrier function and reduced TNF-α-induced barrier compromise, mediated in part by changes in the tight junctional complex.

## Introduction

It is well established in the published literature that compromise of epithelial barrier function is intricately involved in the etiology of a wide range of diseases (Guttman and Finlay, 2009; Torres-Flores and Arias, 2015; DiGuilio et al., 2022). From cancer to chronic inflammation to a diverse spectrum of infectious diseases, even dust mite rhinitis, a barrier leak at the level of the tight junctional (TJ) complex can play a fundamental role in the disease process (Soler et al., 1999; Wan et al., 1999; Guttman and Finlay, 2009; Noureddine et al., 2022). A vibrant and rapidly growing body of literature also exists now, documenting those micronutrients—beneficial compounds and minerals that occur naturally in the diet at very low concentrations—that can improve barrier function and/or protect barrier function from the action of disease modifiers (Amasheh et al., 2009; DiGuilio et al., 2022). Both actions typically ensue at micronutrient concentrations that are above recommended dietary allowance (RDA) levels or, in the case of cell culture models, levels above those found in the culture medium with 10% serum (DiGuilio et al., 2022).

Flavonoids are polyphenolic phytochemical compounds found in plants and are some of the most beneficial micronutrients in our diet. In addition to their well-known role as antioxidants, flavonoids have also become recognized as being very adept at tight junction regulation and epithelial barrier improvement, actions that cannot be attributed simply to their antioxidant capability (Suzuki and Hara, 2011). Flavonoids are also well-described inhibitors of pro-inflammatory cytokine synthesis and secretion (Leyva-López et al., 2016; Martínez et al., 2019; Al-Khayri et al., 2022), actions that would certainly augment, if not account for, their improvement of epithelial barrier function.

Quercetin is one of the most widely abundant flavonoids in fruits and vegetables and, consequently, one of the most abundant in our diet (Formica and Regelson, 1995). It is also one of the most investigated flavonoids with respect to tight junctions and epithelial barrier function. Quercetin has been shown to support epithelial barrier function in a variety of epithelial cell culture models: rat intestinal IEC-6 cell layers (Fan et al., 2021; Fan et al., 2022), human retinal RPE cell layers (Li et al., 2021), human intestinal Caco-2 cell layers (Carrasco-Pozo et al., 2013; Valenzano et al., 2015; Sharma et al., 2020), human intestinal HT29/B6 cell layers (Amasheh et al., 2012), human gingival Gie-3B11 cell layers (Rybakovsky et al., 2017), human umbilical vein ECV304 cell layers (Chuenkitiyanon et al., 2010), and porcine renal LLC-PK1 cell layers (Mercado et al., 2013). In addition to epithelial cell culture models, quercetin also exhibited barrier improvement in epithelial tissue models: hen intestinal tissue (Amevor et al., 2022), pig intestine (Xu et al., 2021), rat lung (Tripathi et al., 2021), and rat ileum and colon (Amasheh et al., 2012). However, it is worth noting that quercetin has been reported to decrease the transepithelial electrical resistance (TER) of dog kidney MDCK II cell layers (Nakashima et al., 2020), although the opposite was reported by Gamero-Estevez et al. (2019), who showed increased transepithelial electrical resistance (TER) along with increased claudins-3 and -4 but decreased claudin-2. A quercetin-induced decrease in TER and an increase in mannitol leak were also reported in a human airway (16HBE) model (Callaghan et al., 2020).

When asserting that quercetin supports epithelial barrier function, it is essential to distinguish between two aspects of that action. The first is that quercetin improves basal-state barrier function, i.e., quercetin acts to increase TER and/or decrease leak of paracellular probe molecules in epithelial cell layers without being exposed to any other agent (Mercado et al., 2013; Valenzano et al., 2015; Rybakovsky et al., 2017; Gamero-Estevez et al., 2019). The second is that quercetin can protect an epithelial cell layer from the compromising effects of substances known to make these cell layers leaky. Quercetin has been shown to successfully protect against leaks induced by acrylamide (Fan et al., 2022), aging (Amevor et al., 2022), microcystin (Zhou et al., 2021), hypoxia (Tripathi et al., 2021), peroxide (Chuenkitiyanon et al., 2010; Li et al., 2021), indomethacin (Carrasco-Pozo et al., 2013; Fan et al., 2021), hyperglycemia (Sharma et al., 2020), and tumor necrosis factor (Amasheh et al., 2012).

This study sought to investigate, for the first time, the epithelial barrier improvement and protection of the Calu-3 airway epithelial model by quercetin. Barrier improvement was recorded by both TER increase and ^14^C-D-mannitol leak decrease, as well as a remodeling effect on TJ proteins. Quercetin’s inhibition of DNA synthesis, leading to a decrease in cell replication in the Calu-3 cell layer, may also be a mechanism of barrier improvement through the resulting decrease in linear junctional density. Protection by quercetin from the barrier compromise induced by the cytokine TNF-α is also shown, a phenomenon that appears to be associated with decreased ERK phosphorylation and a dramatic decrease in the TJ protein claudin-5.

## Materials and methods

### Cell culture

The Calu-3 cell culture, an epithelial cell line derived from human lung adenocarcinoma, was obtained from ATCC (Manassas, VA) and used for 20 weeks before returning to frozen cell stocks. Upon confluence, cells were passaged on a weekly basis by trypsinization [0.25% trypsin and 2.2 mmol/L EDTA (Corning Cellgro, Manassas, VA)]. Cells were seeded at a density of 3.5 × 10^6^ cells/Falcon 75-cm^2^ culture flask with 25 mL of MEM (Corning Cellgro, Manassas, VA), supplemented with 2 mmol/L L-glutamine and 10% fetal bovine serum (Seradigm, VMR Inc., Radnor, PA). Cultures were incubated at 37°C in a 95% air/5% CO_2_ atmosphere.

### Transepithelial electrophysiology and paracellular permeability measurements

Cells were seeded into sterile Millicell polycarbonate (PCF) cell culture inserts [12 mm diameter with 0.4 µm pore size (EMD Millipore, Burlington, MA)] on day 0 at a seeding density of 1.5 × 10^5^ cells/insert. PCF inserts were placed in 24-well cell culture plates. On day 1, all cell layers were refed (0.4 ml apical and 0.6 ml basal–lateral) with the control medium containing 50 U/ml penicillin and 50 μg/ml streptomycin (Corning Cellgro, Manassas, VA). The same refeed procedure was performed on days 4 and 7. Treatments with quercetin and TNF-α began on day 8 post-seeding (when the cell layer barrier was established).

Cell layers were refed with the control or treatment medium on the day of the experiments and allowed to incubate at 37°C for 90 min prior to electrophysiological readings. TER was measured using an epithelial volt/ohmmeter [EVOM (Torres-Flores and Arias, 2015)] with STX2 “chopstick” electrodes (World Precision Instruments, LLC, Sarasota, FL). The resistance of a blank PCF insert was subtracted from each experimental value. These corrected resistances were multiplied by cell layer area (0.6 cm^2^) to achieve ohms x cm^2^.

Following TER measurements, the basal–lateral medium was removed and replaced with 0.6 ml of medium containing 0.1 mM, 0.25 μCi/ml ^14^C-D-mannitol (Perkin-Elmer, Boston, MA) or 10 μM, 0.1 μCi/ml ^14^C-D-inulin (Perkin-Elmer, Boston, MA) and incubated at 37°C. Triplicate 50 µl samples were taken from the basal-lateral medium to determine the specific activity via liquid scintillation counting (LSC). 150 μl samples were taken from the apical medium for LSC at either 60 or 90 min to determine transepithelial flux rates. The flux rate (in picomoles/min/cm^2^) was calculated for the previously mentioned probes diffusing across the cell layer.

### Treatment with TNF-α and quercetin

Quercetin (Sigma-Aldrich, St. Louis, MO) was added directly to the culture medium to a concentration of 400 µM, followed by warming the medium to 38°C–40°C for 30 min with constant stirring. Control conditions likewise had their medium warmed and stirred for 30 min. TNF-α (Peprotech Inc., Rocky Hill, NJ) was prepared as a stock solution (100 ng/μl) in the culture medium and then added to the culture medium to a final concentration of 150 ng/ml. The TNF-α medium was warmed prior to the addition of TNF-α. TNF-α stocks were kept frozen at −80°C and thawed only once. Treatments were applied to the apical and basal–lateral cell surfaces.

### DNA determinations and ^14^C-thymidine incorporations

For determining total DNA, cell layers on their supporting Millicell polycarbonate filters were rinsed five times with 0.154 M NaCl and then immersed in 0.4 M perchloric acid (PCA). After 60 min at room temperature, samples were vortexed and centrifuged at 10,000 g at 4°C. The supernatant was discarded. The pellet was resuspended in 1 M PCA, followed by hydrolysis at 78°C for 45 min. Vortexed samples were then reacted with diphenylamine for spectrophotometric measurement of total DNA (600 nm) (Burton, 1956).

For measurement of thymidine incorporation into DNA, cell layers on Millicell filters were incubated for 3 h at 37°C in complete culture medium containing 0.5 μCi/ml of ^14^C-thymidine (Perkin-Elmer, Boston, MA). Cell layers were then rinsed and harvested in 0.4 M PCA, as previously described. After DNA hydrolysis at 78°C, when samples were taken for spectrophotometric analyses with diphenylamine, additional samples were taken for LSC. Results were expressed as cpm of ^14^C-thymidine/µg of total DNA.

### Linear junctional density measurements

Glass coverslips measuring 1 cm^2^ were sterilized in absolute ethanol and placed in 9.6 cm^2^ (per well) six-well plates (Corning, Kennebunk, ME). 3 × 10^6^ Calu-3 cells were seeded per 9.6 cm^2^ well in complete culture medium. A day before confluence on the coverslips, cell layers were treated with 400 µM quercetin medium (or control medium). After 48 h, the medium was aspirated, and cell layers were rinsed three times with 0.154 M NaCl. Cells were fixed in freshly diluted 4% formaldehyde (in PBS) for 15 min at room temperature and then rinsed three times with PBS. After overnight storage at 4°C, cells were incubated for 10 min in 0.1% Triton X-100 (in PBS), followed by 10% goat serum (Jackson ImmunoResearch Laboratories Inc., West Grove, PA) in PBS for 45 min. Cells were then exposed to a 1:200 dilution of a rabbit polyclonal anti-ZO-1 (Jackson ImmunoResearch Laboratories Inc., West Grove, PA) in 1% goat serum for 1.5 h at room temperature with slow rotation. Cell layers were then rinsed three times with PBS, followed by exposure to anti-rabbit-CY3 antisera (Jackson ImmunoResearch Laboratories Inc., West Grove, PA) in 1% goat serum for 1 h in the dark. Cell layers were then washed 3X with PBS in the dark and then 1X with distilled water, also in the dark. Coverslips were then mounted on slides. Cell layers were photographed at ×400 magnification using a Nikon A1 HD25 confocal microscope (Melville, NY), with images obtained using a Nikon NIS Elements Viewer.

Following the methods of Rabito (1986), linear junctional density was determined by physically tracing cell borders on printed images of ZO-1-immunostained cell layers (100 × 100 microns) (control vs. quercetin-treated cell layers) using a Model 6135 Digital Planimeter (Calculated Industries, Carson City, NV). Measurements were taken on four randomly selected 100 × 100 micron areas for each treatment group. The results were expressed as microns of junctional length per micron of cell layer area (Torres-Flores and Arias, 2015).

### PAGE and Western immunoblots

Cell layers were harvested from Millicell PCF inserts after washing five times with cold saline. 600 μL of ice-cold Buffer A (with protease and phosphatase inhibitors) was then added to each PCF. The cell layer was then physically scraped off the filter at 4°C. The resulting suspension was collected, flash-frozen, and stored at −80°C. Once thawed, lysates were prepared by sonication and ultracentrifugation. Sonication was performed using the Fisher Scientific Sonic Dismembrator (Model 100), Setting 3. Ultracentrifugation was performed in a Beckman Model L-80 with a Ti-70 rotor at 39,000 rpm for 1 h at 4°C. After this first ultracentrifugation, the supernatant (cytosolic fraction) was discarded, followed by solubilization of the pellet (membrane/cytoskeletal fraction) in an SDS lysis buffer. Samples of these detergent-soluble lysates were analyzed by PAGE using a 10%–20% gradient Tris–glycine gel (Invitrogen, a division of Thermo Fisher Scientific) at 120 V for 80 min. Precision Plus Kaleidoscope Protein Standards (Bio-Rad Inc., Hercules, CA) were included in each gel. Proteins were transferred at 30 V for 1 h from the gel to a nitrocellulose membrane. The membranes were then washed three times with PBS-T (0.3% Tween 20) for 10 min and blocked with 5% milk/PBS-T at room temperature for 1 h. Membranes were incubated with the specific primary antibodies [anti-claudin-1, -3, -4, -5, and -7, tricellulin, or occludin (Thermo Fisher Scientific) and anti-claudin-2 (Abcam, Cambridge, MA)] at 0.5 μg/mL in 5% milk/PBS overnight at 4°C. pERK (Cell Signaling) and MAPK-ERK (Thermo Fisher) were used at a 1:1000 dilution. The membranes were washed again three times, 10 min each, with PBS-T, and then incubated with the secondary antibody (rabbit anti-mouse- or goat anti-rabbit-IgG antibody labeled with horseradish peroxidase (Southern Biotech, Birmingham, AL) for 1 h at RT. The membranes were then washed again four times, 10 min each, with PBS-T, and treated for 10–60 s with Western Lightning Plus-ECL chemiluminescence reagents (Perkin-Elmer). The membranes’ protein band densities were quantified using the Bio-Rad ChemiDoc Imaging System. The band densities of the experimentally treated cell samples were compared to the averages of the corresponding control cell samples.

### Statistics

All data are expressed as the mean ± standard error of the mean with the number of replicates provided for each set of studies. Statistical significance in these studies was determined by the mean of two-sided Student’s t tests. Significance was claimed when *p* < 0.05.

## Results

**FIGURE 1:**
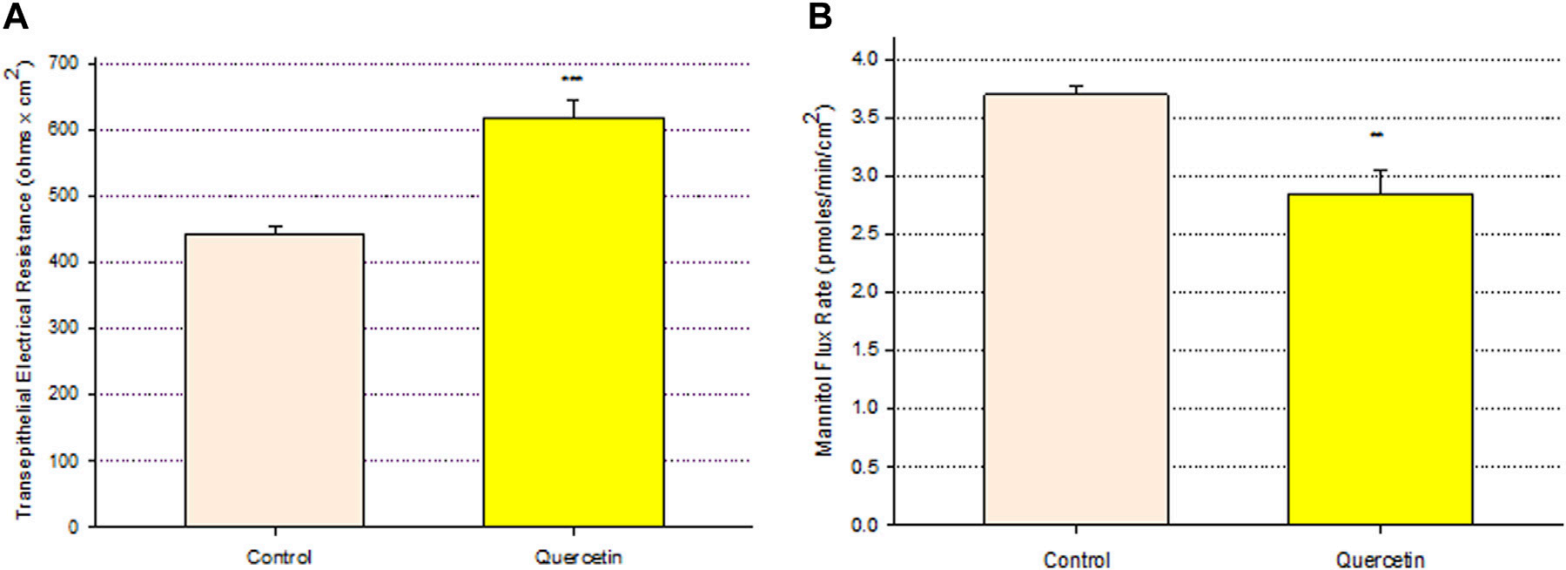
Effect of 400 μM quercetin on Calu-3 cell layer barrier function. **(A)** TER and **(B)** transepithelial mannitol flux rate were measured as described in Materials and Methods, 72 h after quercetin treatment. Data are represented as mean ± standard error for *n* = 5 cell layers per condition. **p < 0.01 and ***p < 0.001 (Student’s t-test, two-tailed).

**FIGURE 2:**
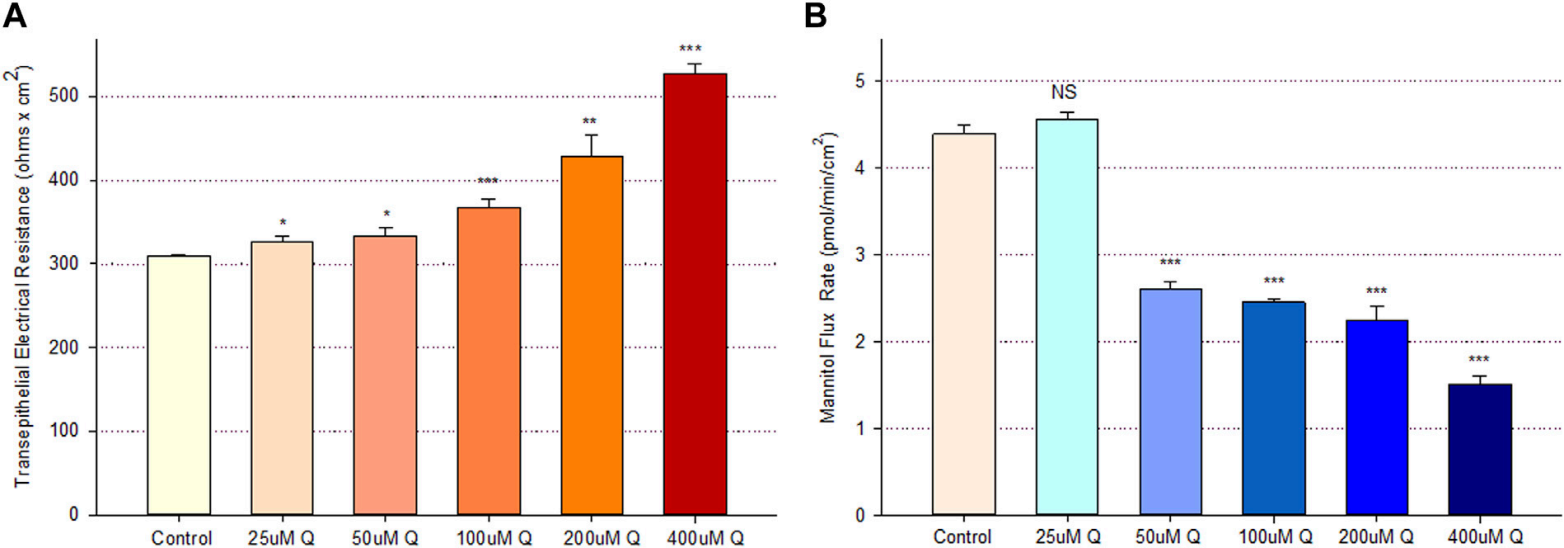
Concentration dependence of the quercetin effect on Calu-3 cell layer barrier function. **(A)** TER and **(B)** transepithelial mannitol flux rate were measured as described in Materials and Methods, 48 h after quercetin treatment. Data are represented as mean ± standard error for *n* = 6 cell layers per condition. *p < 0.05, **p < 0.01, and ***p < 0.001 vs. control. NS indicates non-significance (Student’s t-test, two-tailed).

Treatment of Calu-3 cell layers with 400 µM of quercetin for 72 h resulted in a statistically significant increase of over 40% in TER and a simultaneous significant decrease of over 25% in ^14^C-D-mannitol transepithelial diffusion (Figure 1). The simultaneous occurrence of both events strongly suggests that quercetin was causing a decrease, specifically in the paracellular permeability of Calu-3 cell layers. This quercetin-induced improvement of barrier function was concentration-dependent, with activity observed at concentrations as low as 25–50 µM (Figure 2).

**FIGURE 3:**
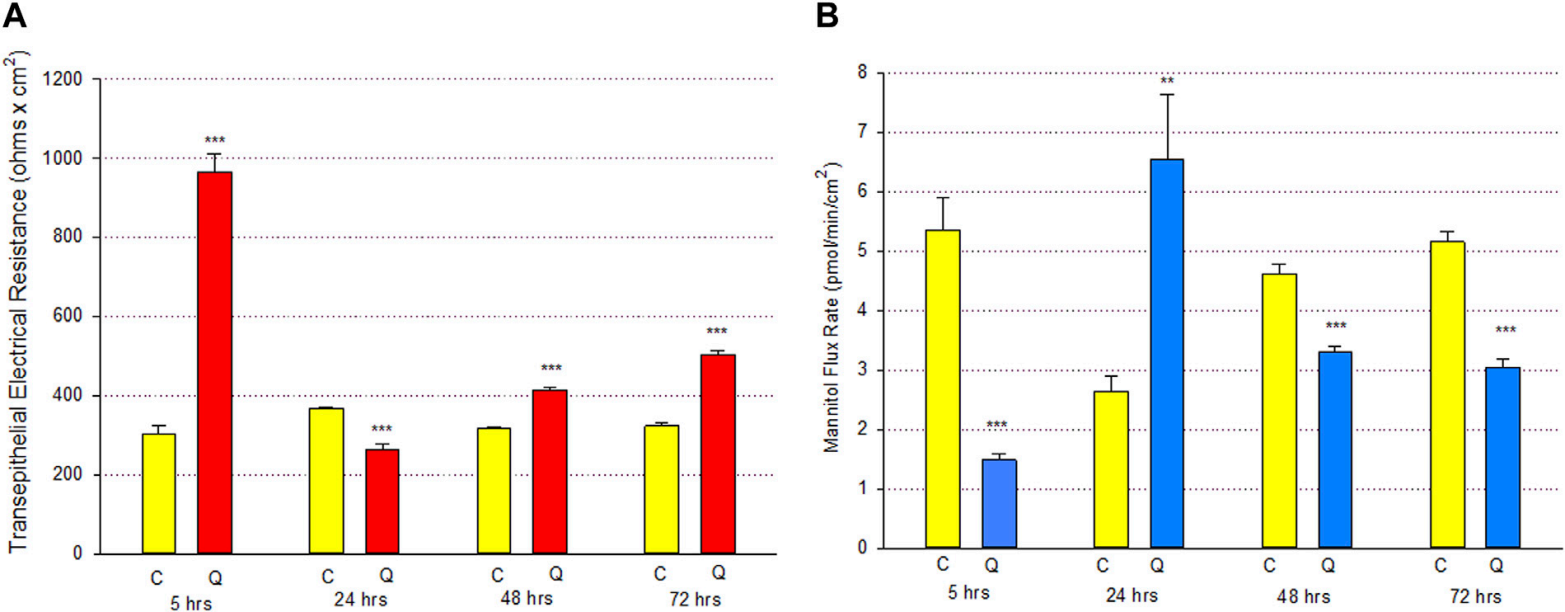
Time course of 400 µM quercetin effects on Calu-3 cell layers. **(A)** TER and **(B)** transepithelial mannitol flux rate were measured as described in Materials and Methods at various time points following treatment with 400 µM quercetin. *n* = 6 cell layers per time point. **p < 0.01 and ***p < 0.001 vs. time-matched control (Student’s t-test, two-tailed). C indicates control. Q indicates quercetin.

The time course of quercetin’s (400 µM) action on Calu-3 barrier function was unusual. The dramatic improvement in barrier function, as evidenced by an over 3-fold increase in TER and a 70% decrease in ^14^C-D-mannitol leak, was seen as early as 5 h post-treatment. By 24 h, this situation reversed completely, with a 30% highly significant decrease in TER and an over 150% increase in ^14^C-D-mannitol leak compared to time-matched controls. At 48 h post-exposure, the situation spontaneously reversed itself again, with a 30% highly significant increase in TER and a 35% decrease in ^14^C-D-mannitol leak. The barrier then tightened further by 72 h on both metrics, all this occurring with only one treatment with quercetin (Figure 3).

**FIGURE 4:**
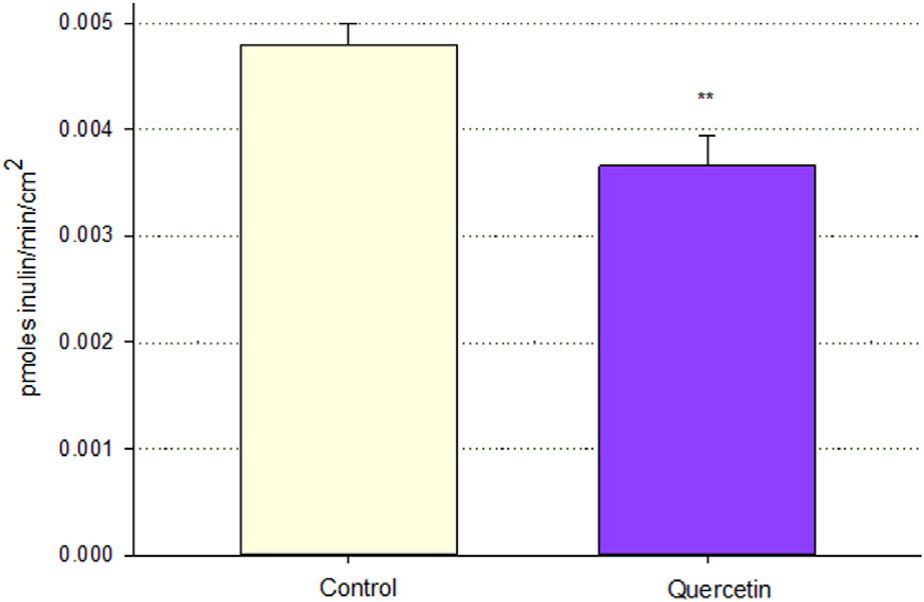
Effect of 400 µM quercetin on transepithelial diffusion of ^14^C-inulin across Calu-3 cell layers. The transepithelial flux of ^14^C-inulin was measured as described in Materials and Methods, 72 h after 400 µM quercetin treatment. Data are represented as mean ± standard error for *n* = 5 cell layers. **p < 0.01 vs. control cell layers (Student’s t-test, two-tailed).

The tightening of the barrier did not apply to only small solutes. Increased TER would be indicative of decreased paracellular conductance of Na^+^ and Cl^−^ ions, whose molecular weights are 23 and 35, respectively. The decreased permeability to D-mannitol is for a 182 molecular weight solute. However, Figure 4 shows that a 72-h treatment of Calu-3 cell layers with 400 µM quercetin also decreased the (paracellular) leak of ^14^C-inulin—a 3,500 mw solute. This signifies that the quercetin-treated barrier is likely tightening to mono- and di-saccharides, amino acids, peptides, and small proteins, as well as salts and water.

**FIGURE 5:**
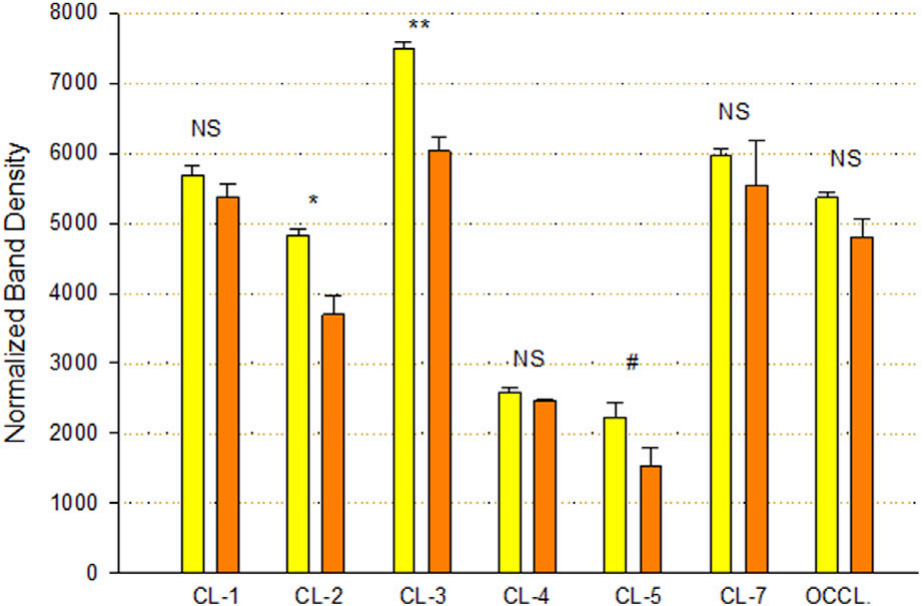
Effect of 400 µM quercetin on Calu-3 cell layer tight junctional proteins in detergent-soluble fractions. Data shown represent densitometry of Western immunoblots of various TJ proteins of cell layers treated with 400 µM quercetin for 72 h. Data are for TJ protein abundance in the particulate fraction. Band densities of immunoblot samples were quantified as described in Materials and Methods. Bars represent the mean ± standard error for *n* = 3 cell layers. *p < 0.05, **p < 0.01, and NS indicates non-significance (Student’s t-test, two-tailed). ^#^ *p* < 0.05 (Student’s t-test, one-tailed). Yellow bars indicate control. Orange bars indicate quercetin.

An analysis of TJ proteins by PAGE and Western immunoblot of Calu-3 cell layers treated with 400 µM qQuercetin for 72 h showed statistically significant changes in several TJ proteins in particulate (detergent-soluble) fractions (Figure 5). Specifically, we observed a statistically significant 25% decrease in claudin-2, a 20% decrease in claudin-3, and a borderline significant (*p* < 0.05, one-tailed Student’s t-test) 35% decrease in claudin-5 because of quercetin treatment. Claudin-1, -4, and -7 and occludin abundance were not significantly changed.

**FIGURE 6:**
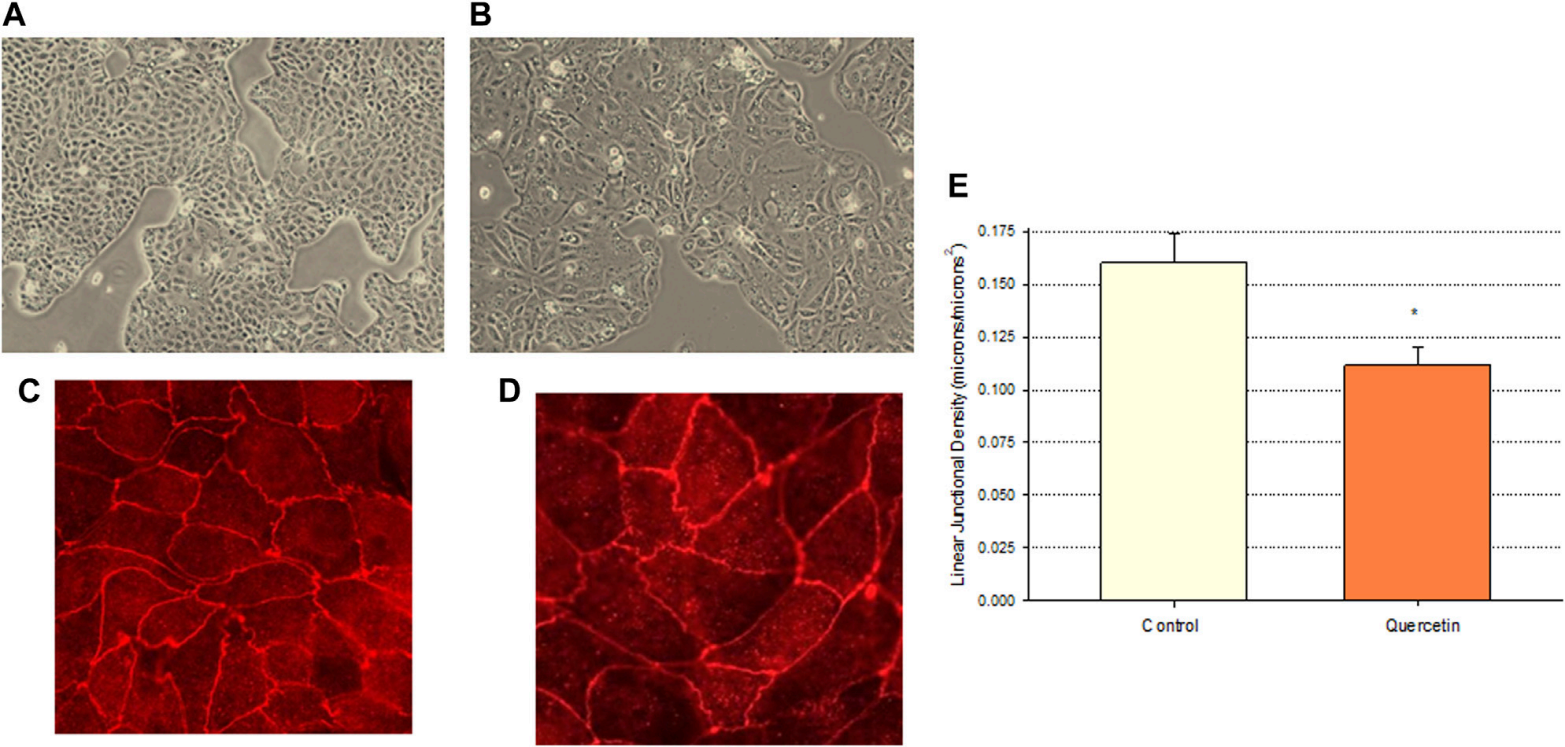
Effect of 400 µM quercetin on the apparent cell size and linear junctional density of Calu-3 cell layers. 24 h after being seeded on glass coverslips, cell layers were treated with 400 µM quercetin for 48 h. Cell layers were then photographed at 100X using an inverted phase-contrast microscope, fixed, and immunostained with a primary antibody to ZO-1 and a rhodamine-conjugated fluorescent secondary antibody as described in Materials and Methods. Cell layers were then photographed at 100X. **(A, B)** Phase-contrast micrographs of sample control **(A)** and quercetin-treated **(B)** cell layers. **(C,D)** ZO-1 immunofluorescent micrographs of control **(C)** and quercetin-treated **(D)** cell layers. **(E)** Quantitative comparison of the linear junctional density of four control and four quercetin-treated cell layers, as measured by using a linear planimeter. Bars represent the mean ± standard error of junctional density for *n* = 4 cell layers. *p < 0.05 (Student’s t-test, two-tailed).

**FIGURE 7:**
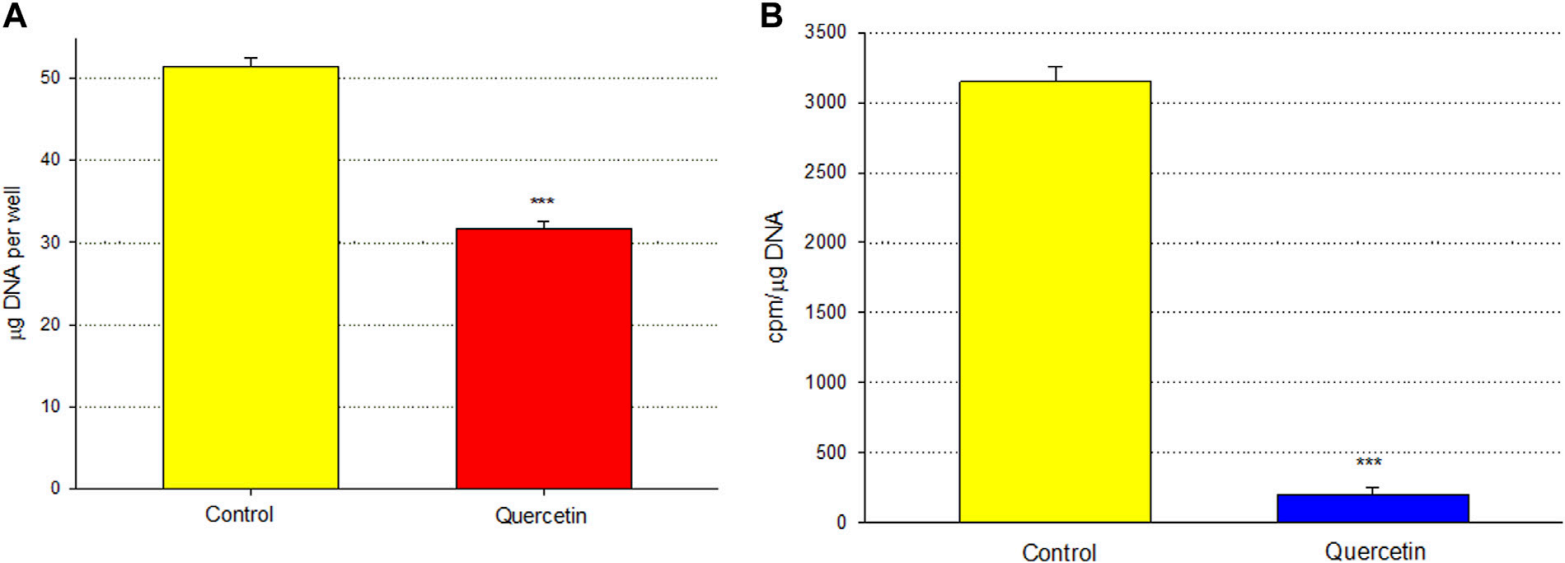
Effect of 400 µM quercetin on total DNA and ^14^C-thymidine incorporation into the DNA of Calu-3 cell layers. As described in Materials and Methods, 2 days after seeding into Millicell PCF filter units, incompletely formed cell layers were treated with 400 µM quercetin for 48 h. Cell layers were incubated with 0.1 μCi/ml ^14^C-thymidine for the final 3.5 h. Cell layers were then harvested, and total DNA **(A)** and ^14^C-thymidine-labeled DNA **(B)** were determined. Bars represent the mean ± standard error for *n* = 4 cell layers. ***p < 0.01 (Student’s t-test, two-tailed).

Calu-3 cultures treated with quercetin were observed to have increased cell size (Figure 6). Of distinct note for a study on barrier function, the increased cell size of the quercetin-treated cell layers translated to a statistically significant decrease in linear junctional density (total cell border length) as compared to matched controls (Figure 6E). This results in less junctional density per unit area of cell layer, and along with the altered tight junctional composition (Figure 5), both these factors can be reasons for the decreased paracellular permeability to probes such as 14C-D-mannitol as well as the increased TER. Proceeding from the microscopy observations, the potential effect of 400 µM quercetin on Calu-3 DNA synthesis was tested in cell layers cultured on permeable filters. Subconfluent cultures treated with quercetin exhibited a statistically significant decrease in DNA synthesis, as evidenced by a reduction in total DNA and a reduction in the incorporation of 14C-thymidine into DNA (Figure 7).

**FIGURE 8:**
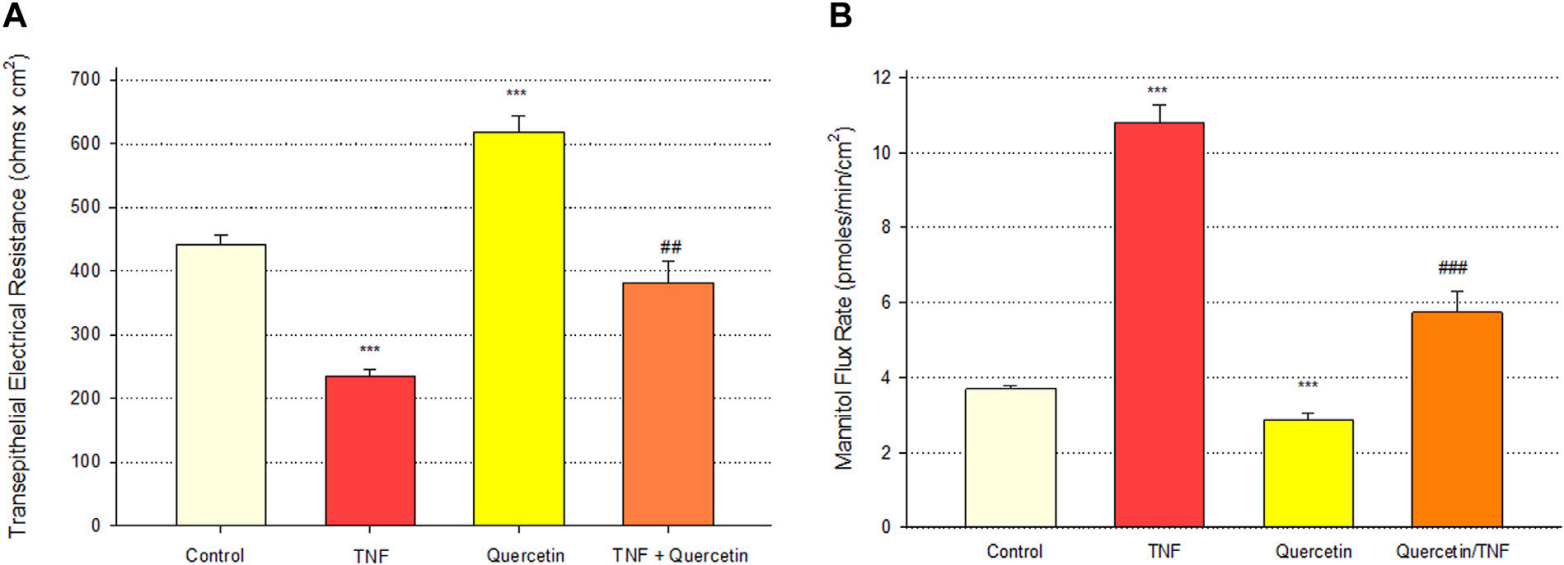
Effect of 400 µM quercetin on 150 ng/ml TNF-α induced Calu-3 transepithelial leak. **(A)** TER and **(B)** transepithelial mannitol flux rate were measured as described in Materials and Methods, 72 h after simultaneous treatment with TNF-α and/or quercetin. Bars represent the mean ± standard error for *n* = 5 cell layers [except for the combination of TNF-α and quercetin (*n* = 3)]. ***p < 0.001 vs. control, ^##^ *p* < 0.01 vs. TNF-α-treated condition, and ^###^ *p* < 0.001 vs. TNF-α-treated condition (Student’s t-test, two-tailed).

**FIGURE 9:**
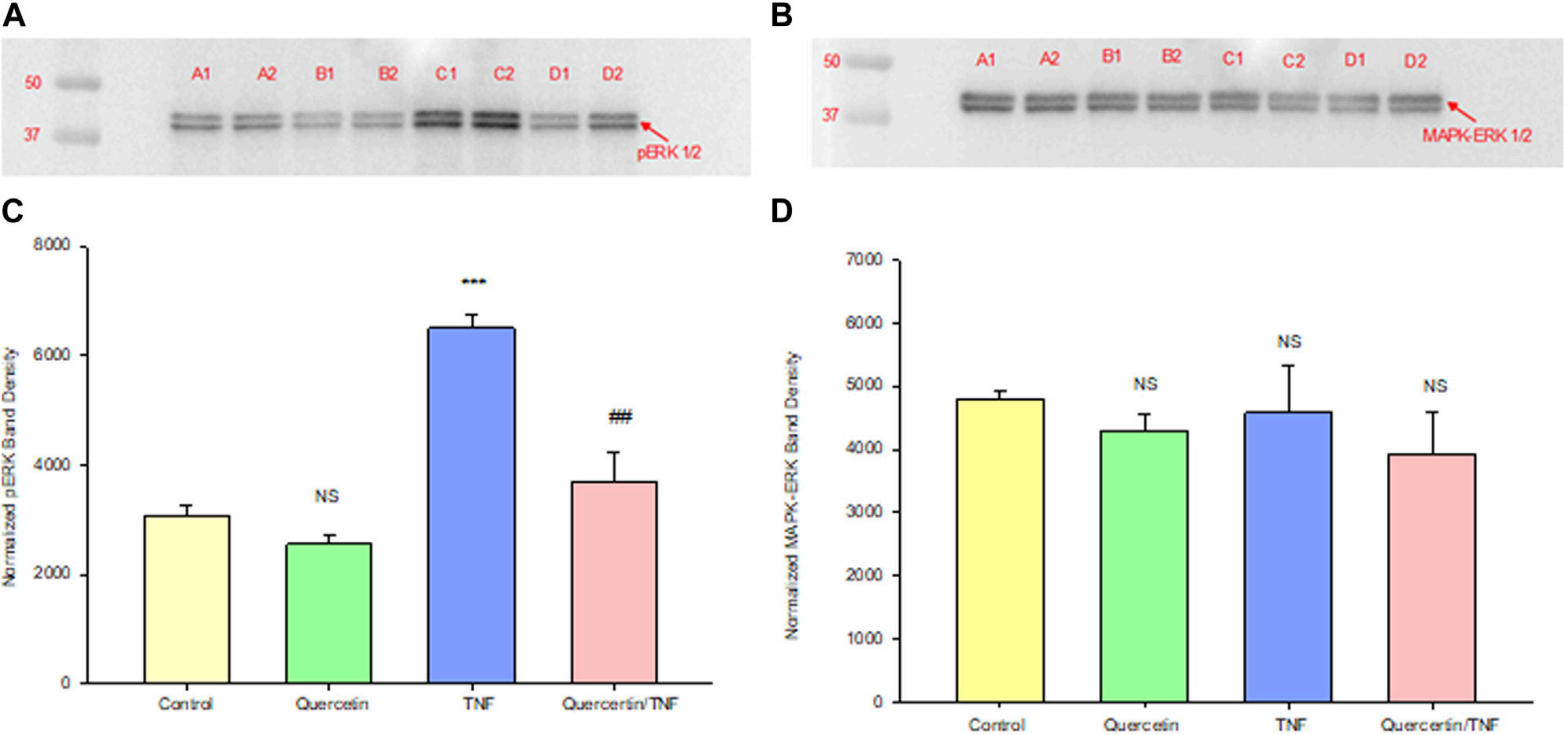
Effect of 150 ng/ml TNF-α or TNF-α + 400 µM quercetin on phosphorylated ERK-1,2 levels in Calu-3 cell layers. Confluent cell layers were treated with control or a quercetin-containing medium for 2.5 h prior to treatment with TNF-α or TNF-α + quercetin for 45 min. Cell layers were harvested and lysed, and PAGE and Western immunoblots were performed for phosphorylated ERK-1/2 **(A)** and total MAPK- ERK-1/2 **(B)**. Lanes A1–A2: control; lanes B1–B2: quercetin-treated; lanes C1–C2: TNF-α-treated; and lanes D1–D2: TNF-α/quercetin-treated. Two out of three immunoblot lanes are shown. Band densities from all three cell layers were quantified as described in Materials and Methods **(C, D)**. Bars represent the mean (band density) ± standard error for three cell layers. ***p < 0.001 vs. control and ^##^ *p* < 0.01 vs. TNF-α-treated. NS, not significant vs. control cell layers (Student’s t-test, two-tailed).

**FIGURE 10:**
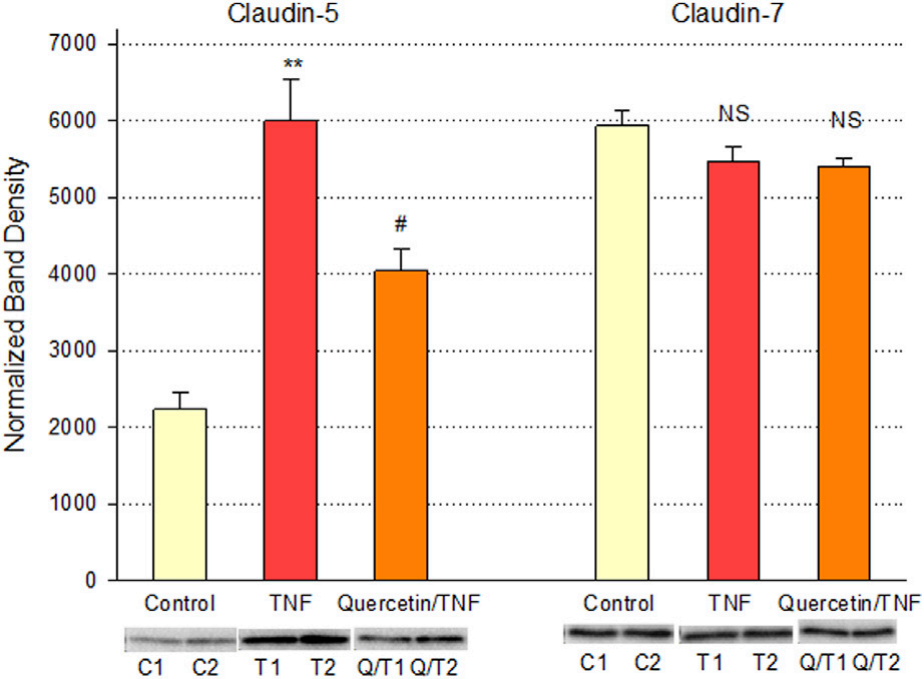
Effect of 400 µM quercetin on TNF-α’s effect on Calu-3 cell layer claudin-5 and -7 tight junctional proteins. Data shown represent densitometry of Western immunoblots of the TJ proteins claudin-5 and -7 in detergent-soluble fractions of cell layers treated with 150 ng/ml TNF-α ± 400 µM quercetin for 72 h. Data shown represent claudin protein abundance in the particulate fraction. Band densities of immunoblot samples were quantified as described in Materials and Methods. Bars represent the mean ± standard error for *n* = 3 cell layers. **p < 0.01 vs. control and ^#^ *p* < 0.05 vs. TNF-α. NS indicates non-significance (Student’s t-test, two-tailed).

Quercetin was effective not only in improving the barrier function of normal, control Calu-3 cell layers but also in reducing the barrier compromise caused by the pro-inflammatory cytokine TNF-α. 150 ng/ml of TNF-α reduced TER by approximately 50% while simultaneously increasing 14C-D-mannitol leak by approximately 40% (Figure 8). 400 μM quercetin significantly reduced these actions of TNF-α when TNF-α and quercetin were simultaneously presented. The transepithelial leak produced by TNF-α may have been transduced by dramatically increased ERK 1/2 phosphorylation; this increased phosphorylation was totally inhibited by 400 µM quercetin (Figure 9). The barrier compromise caused by 72-h exposure to TNF-α coincided with a dramatic (over 200%) increase in the tight junction protein, claudin-5. This increase was significantly reduced by simultaneous exposure to 400 µM quercetin (Figure 10).

## Discussion

The role of flavonoid micronutrients in improving epithelial barrier function and ameliorating the barrier compromise/leak caused by pro-inflammatory cytokines is becoming increasingly established in the published literature (Amasheh et al., 2008; Suzuki and Hara, 2009; Suzuki and Hara, 2011; Carrasco-Pozo et al., 2013; Valenzano et al., 2015; Zhang et al., 2022). The flavonoid, quercetin, and airway epithelial barriers, such as Calu-3, are mere specific examples of an overall flavonoid trend regarding this class of polyphenols improving barrier function. In the series of experiments presented in this study, we add to that body of evidence of barrier improvement and protection by flavonoids (Figure 1). This activity is shown to be concentration-dependent (Figure 2) and exhibits a curious, biphasic temporal response (Figure 3) that has also been previously reported in MDCK renal epithelia (Gamero-Estevez et al., 2019). It encompasses improvement of normal (control) epithelial barrier function and protection from the effects of a leak inducer (Figure 8).

The clinical compromise of airway barrier function—and resultant lung water accumulation—was evident in dramatic fashion very recently in the COVID-19 pandemic and the significant need that it generated for critical care medicine and mechanical ventilators (Rasch et al., 2021; Virot et al., 2021). However, those needs are equally relevant in acute respiratory distress syndrome (Kreuzfelder et al., 1988). Both instances become compelling reasons to search out and research therapeutics for barrier compromise. The fact that naturally occurring—even dietary—compounds are very adept in that regard offers a safety and economic attribution that is very appealing. Although not a flavonoid or polyphenol, vitamin D offers yet another example of a dietary compound that proved clinically useful in COVID-19 and exhibited the ability to modify and improve the barrier function of airway epithelial models (Bae et al., 2022; Rastogi et al., 2022; Gao et al., 2023; Rybakovsky et al., 2023).

Among researchers who study barrier remodeling, it is always tempting to think that induced TJ remodeling is the reason for the ability of a particular agent, such as quercetin, to improve barrier function. The TJ protein changes observed in this study and reported in Figure 5 (such as decreased claudins-2, -3, and -5) may indeed be evidence of such activity. However, simpler explanations may also be available, and the observations that quercetin inhibits cell replication (Figure 7) and decreases linear junctional density (Figure 6) may be equally important. Rabito (1986) made the original observation in LLC-PK_1_ renal epithelial cell layers that decreased linear junctional density would translate to improved barrier function. Our observation that quercetin can affect ERK 1/2 phosphorylation (Figure 9) represents a more fundamental molecular mechanism consistent with observations by others that inhibiting ERK phosphorylation decreases TJ leaks and cell cycle activity (Crews et al., 1992; Kevil et al., 2000; Petecchia et al., 2012). Therefore, TJ remodeling and the decreased linear junctional density resulting from increased cell size (both mediated by changes in ERK 1/2 phosphorylation) become viable potential mechanisms to explain the improved Calu-3 barrier function seen here.

In addition, regarding the molecular mechanism, it is worth noting that flavonoids (e.g., genistein but also quercetin), as a class, exhibit protein kinase C-inhibitory activity (Suzuki and Hara, 2011). Given that protein kinase C activation—including protein kinase C delta—is a highly effective way to increase TJ leak (Mullin et al., 1998a), inhibition of protein kinase C by quercetin could be another means by which quercetin improves Calu-3 barrier function.

Turning to the biomedical implications of these and similar findings, we would point out that a TJ (barrier) leak is intrinsic to a wide variety of diseases and disease processes and that an agent that opposes such a leak would likely have therapeutic potential, a subject covered in a recent extensive review (DiGuilio et al., 2022). Diarrhea, liver insufficiency, renal (tubular) insufficiency, and accumulation of lung water (think multi-organ failure here) are clinical manifestations of the type of barrier leak that we observe in this study (e.g., the TNF-α-induced leak) as revealed by decreased TER (i.e., increased passive transepithelial leak (conductance) of salt and water) and increased ^14^C-D-mannitol permeability (indicative of leak of sugars, amino acids, and other small molecules). The ability of quercetin to partially reduce these aspects of leak suggests its utility in treating those manifestations. However, the quercetin-induced decrease in leak of ^14^C-inulin (Figure 4) indicates that it is not simply physiological osmolytes/substrates and water whose leak is being reduced by flavonoids but also very likely the pathophysiological transepithelial leak of peptides and small proteins that could then result in abnormal regulatory effects in these tissues (Mullin and McGinn, 1987; Mullin et al., 1998b). Therefore, the therapeutic action of naturally occurring leak reducers, such as quercetin, could be biomedically substantial.

In summary, our results demonstrated that quercetin can improve Calu-3 epithelial barrier function and reduce TNF-α-induced barrier compromise, mediated in part by changes in the tight junctional complex. In this study, we add to the knowledge of the regulation of barrier function in a widely used and applicable model of human airway physiology. We also lay out for discussion whether flavonoids such as quercetin could have adjuvant therapeutic utility in airway diseases that involve barrier compromise and fluid accumulation, such as but not exclusive to the recent and ongoing COVID-19 outbreaks.

## Data Availability

The raw data supporting the conclusion of this article will be made available by the authors, without undue reservation.
