# Supplementary figures and images for: Quercetin improves and protects Calu-3 airway epithelial barrier function
Source: Front Cell Dev Biol. 2023 Nov 23;11:1271201. doi: 10.3389/fcell.2023.1271201 (PMC10701405; doi:10.3389/fcell.2023.1271201)

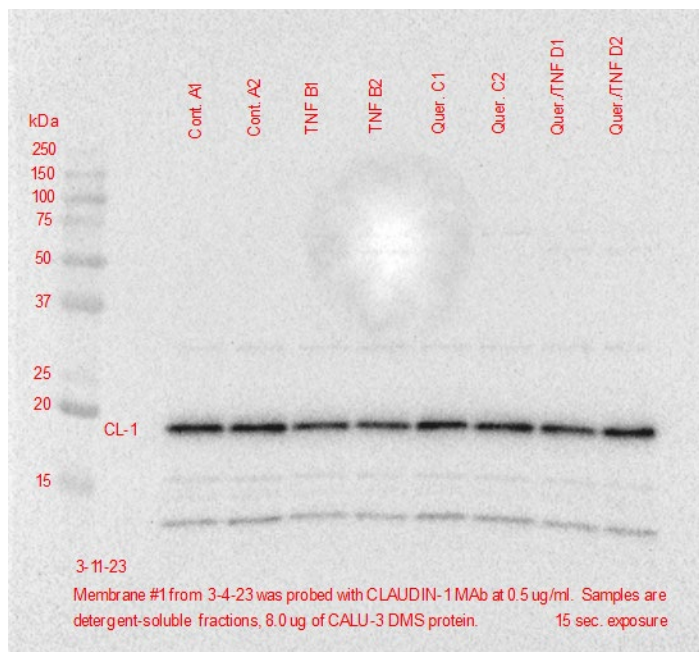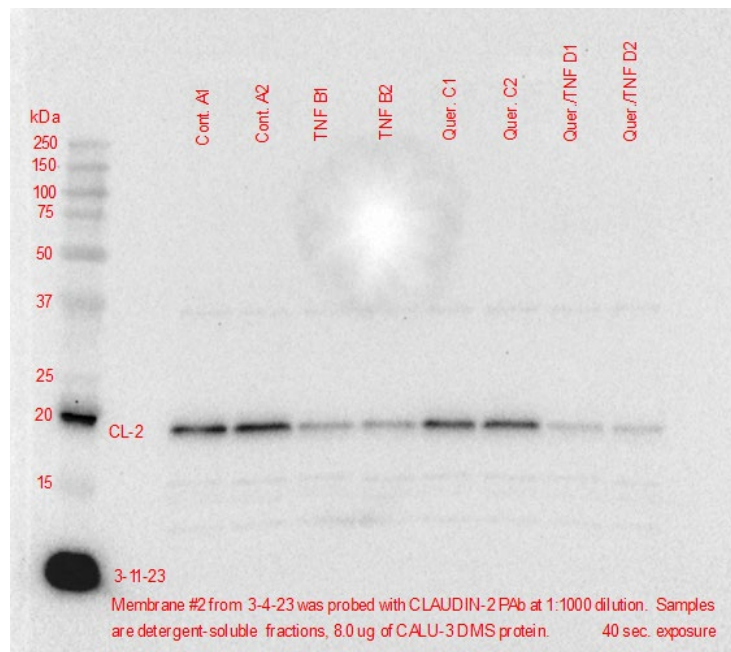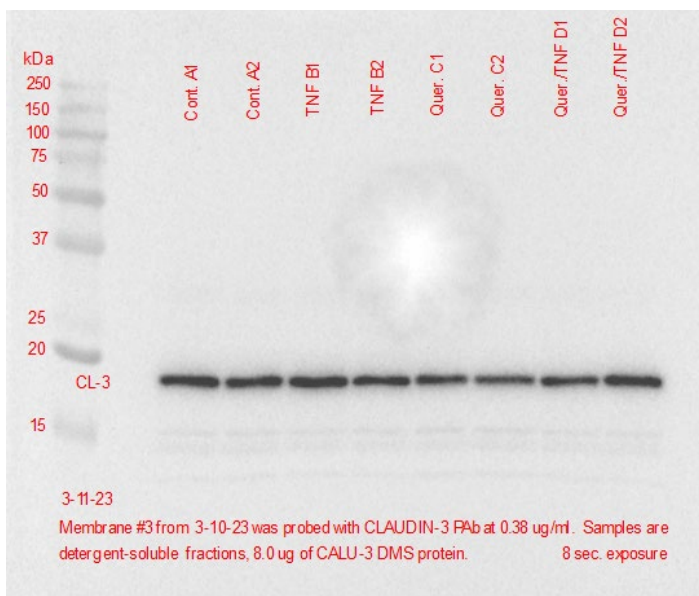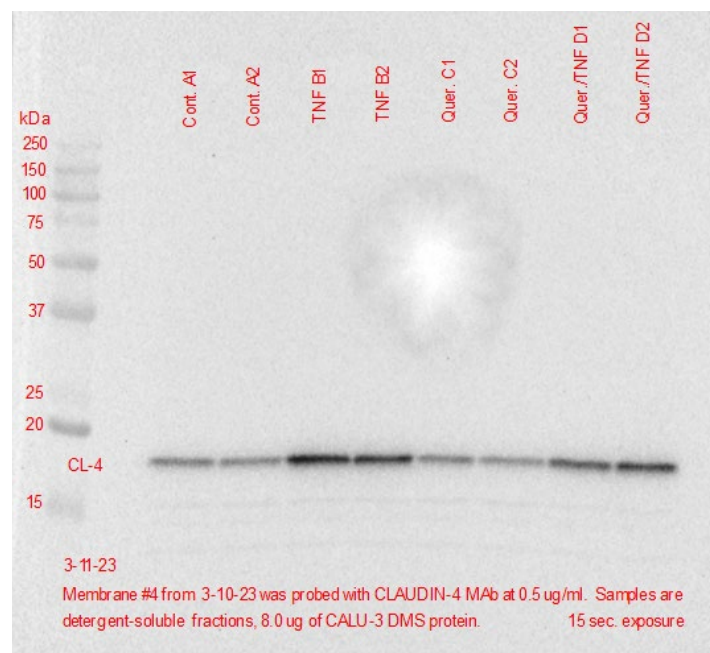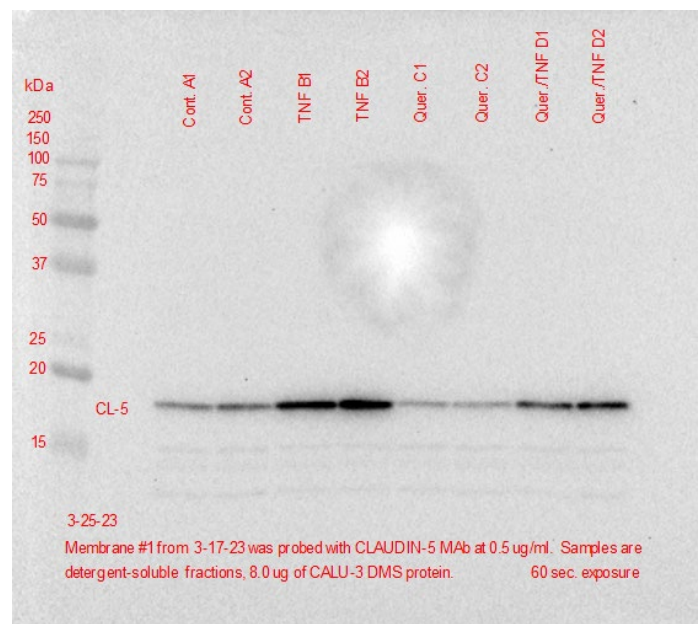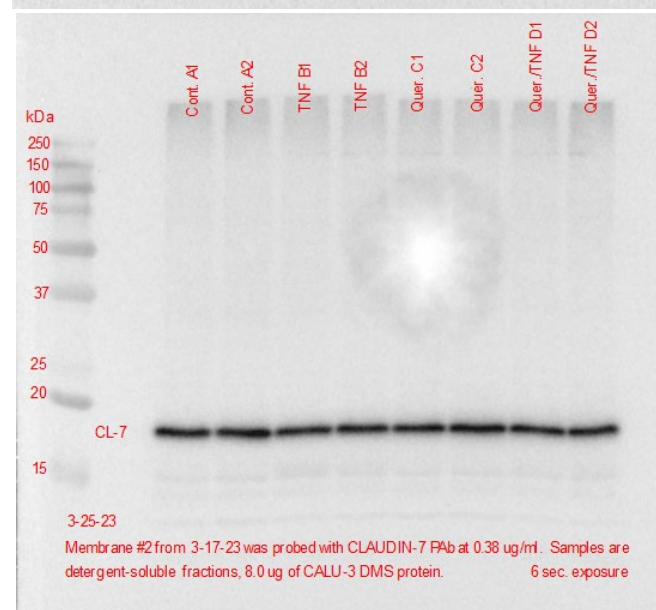

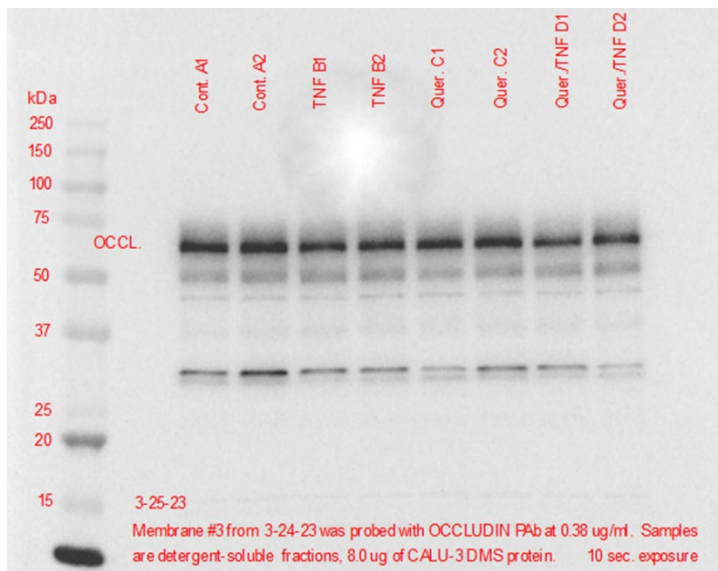

Supplement: Supplementary file 1 [file DataSheet1.PDF]
